# Do Patients With Neck of Femur Fractures (NOFF) Experience Long Pre-Hospital Delays in the United Kingdom?

**DOI:** 10.7759/cureus.45729

**Published:** 2023-09-21

**Authors:** Raja Jambulingam, Alice Campion, Joshua Howard, Devi Prakash Tokala

**Affiliations:** 1 Trauma and Orthopaedics, Royal Gwent Hospital, Newport, GBR

**Keywords:** timing of hip fracture, pre-hospital delay, pre-operative delay, geriatric hip fracture, fracture neck of femur

## Abstract

Background and objective

It is well documented that prolonged preoperative delay is associated with increased morbidity and mortality among patients presenting with neck of femur fractures (NOFF). The target time from arrival to the emergency department (ED) to be transported to the theatre for these patients in England is 36 hours. However, the time before the patient arrives at the hospital is not often considered. In light of this, we aimed to assess the duration of the waiting period for NOFF patients before they are brought to the ED.

Methods

Data were collected retrospectively using IT and theatre systems at a single trust. A total of 223 consecutive NOFF patients undergoing operations in the six-month period between February and August 2020 were reviewed.

Results

The mean time for ambulance response was one hour and 50 minutes, whereas the time spent in the ambulance was one hour and 47 minutes and the total pre-hospital time was three hours and 37 minutes (range: 59 minutes to 14 hours and 41 minutes). The mean time from ED arrival to the theatre was 33 hours and one minute. The mean total preoperative time was 36 hours and 38 minutes.

Conclusion

The mean pre-hospital time of three hours and 37 minutes represents approximately 10% of the 36-hour national target. Pre-hospital time is often overlooked when considering the order of the list for the theatre. It may be possible to reduce morbidity and mortality by prioritising patients with a longer pre-hospital time, especially given our finding that some patients may wait up to 14 hours. We recommend that pre-hospital time be considered for all patients with NOFF.

## Introduction

Neck of femur fractures (NOFF) are among the most common fragility fractures reported in the UK, with an annual incidence of over 75,000 [1]. These fractures are associated with high mortality and morbidity rates among elderly patients, estimated to be around 7-10% at 30 days [2,3]. This presents a major healthcare challenge. Furthermore, providing care for these patients costs over £2 billion per year, leading to a huge economic burden [4-6].

It is well documented that delays in receiving surgery have an adverse effect on morbidity and mortality rates [7-9]. Hence, the National Institute for Health and Care Excellence (NICE) has recommended that operations for NOFF patients be performed on the day of or the day after their admission to the emergency department (ED) [1]. The British Orthopaedic Association (BOA) also recommends that these patients undergo surgery within 36 hours of their arrival at the ED [10]. However, the 36-hour target does not take into account any potential pre-hospital delay. Hence, the aim of this study was to assess the average length of time an NOFF patient may wait before arriving at the ED.

## Materials and methods

This study was conducted from February to August 2020 at a single district general hospital in Wales. Trust theatre systems (“ORMIS”) and clinical IT systems (“Clinical Workstation”) were used to retrospectively collect data. Ethical approval was not sought as the study was retrospective in nature and no patients were physically involved or affected by this study.

The data of all patients who underwent surgery for primary NOFF were included in the initial compilation, but those of some were subsequently filtered out based on our exclusion criteria (Table 1). Polytrauma patients, inpatient fallers, and delayed presentations were excluded, particularly in cases where patients had come via another unit.

**Table 1:** Exclusion criteria

Criteria	Number of patients
Incomplete data	58
Inpatient falls	8
Delayed presentation	11
Polytrauma	2
Total	79

Patient details compiled from the theatre system were used to locate scanned paramedic sheets on the Clinical Workstation. Ambulance response and arrival times to the ED were then recorded from the ambulance sheets. The subsequent theatre arrival time for each patient was taken from the theatre system to calculate the time from ED arrival to the time of arrival to the theatre.

## Results

A total of 302 patients were identified as having undergone hip operations on our theatre operating system during the six-month time frame. After the application of the exclusion criteria, 223 patients were deemed suitable for the final analysis.

The time taken from the initial ambulance call to ambulance arrival was considered the ambulance response time (ART). The time from ambulance arrival at the patient’s home to ED arrival was labelled as ambulance waiting time (AWT). This included time spent waiting in the ambulance outside of the ED. The sum of the ART and AWT was defined as the pre-hospital time (PHT). The subsequent time from arrival to ED to arrival at the theatre was the admission to theatre time (ATT). The sum of the PHT and the ATT was taken to be the total pre-theatre time (TPTT). The average values are shown in Table 2.

**Table 2:** Timings for the patient at various stages of the pre-hospital journey ART: ambulance response time; AWT: ambulance waiting time (time spent in the ambulance); PHT: pre-hospital time; TTT: time to theatre (from arrival at the ED); TPTT: total pre-theatre time (from ambulance pickup)

Results	ART	AWT	PHT	TTT	TPTT
Mean, hours	1:50	1:47	3:37	33:01	36:38
Median, hours	1:03	1:40	2:58	26:29	31:43
Range, minutes	4–803	32–390	59–881	182–8117	283–8885

**Figure 1:**
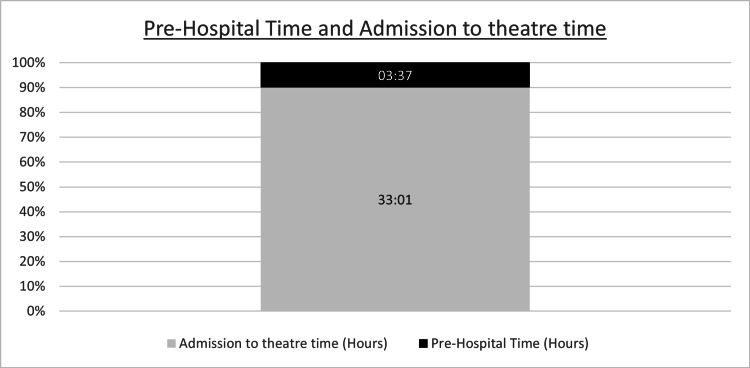
Pre-hospital time expressed as a percentage of time from ED admission to arrival at the theatre ED: emergency department

The mean pre-hospital time was three hours and 37 minutes, representing 9.87% (range: 1.3%-50.8%) of the TPTT, 36 hours and 38 minutes, and 10.95% (range: 1.4%-103%) of the time from ambulance arrival to arrival to the theatre, 33 hours and one minute (Figure 1).

**Figure 2:**
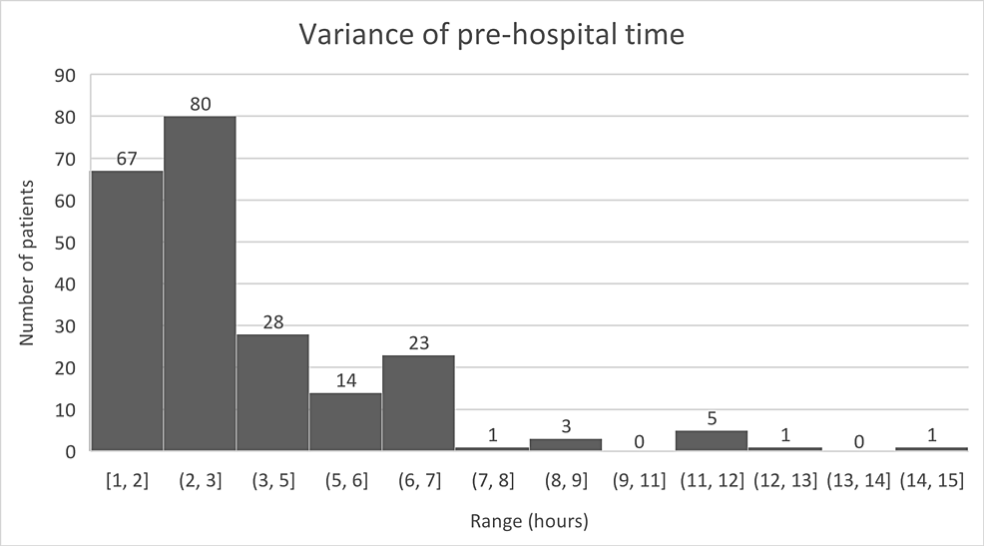
Histogram showing the variance in pre-hospital time (PHT)

In some cases, the pre-hospital time was significantly higher, with one patient waiting 14 hours and 41 minutes. This could represent a much larger proportion of the total pre-theatre time. The variance is demonstrated in the histogram below (Figure 2).

## Discussion

Currently, there is sufficient evidence to suggest that outcome measures such as medical complications, pressure sores, pain control, and length of stay can all be improved by striving to achieve minimal preoperative delay [11,12]. NOFF patients are unable to mobilise, or even stand, following their injury. Therefore, long pre-hospital wait times pose a significant risk of ‘long lie’ and associated conditions including rhabdomyolysis, hypothermia, and pressure sores. Lefaivre et al. demonstrated that pre-theatre delays of just 24 hours significantly increased the risk of minor medical complications, while a delay of 48 hours significantly increased the risk of major medical complications as well as pressure sores [13]. Al-Ani et al. report an increasing chance of developing pressure sores in cases with delays beyond 36 and 48 hours [9].

Hip fractures are usually extremely painful, and patients often require regular opiate-based analgesia while awaiting surgery due to the severity of their pain [13]. Apart from causing a large physical and psychological burden, it is also associated with a range of side effects including delirium, constipation, and urinary retention [14]. However, pain postoperatively is often easier to manage and does not appear to be affected by the length of preoperative delay, but rather by the type of procedure performed [15,16]. The length of hospital stay is also impacted by the timing of surgery, as longer delays to surgery can increase recovery time. Doruk et al. have highlighted the benefits of early surgery in patients with NOFF by demonstrating that delays of greater than five days were associated with increased time to weight-bearing and worse activity-of-daily-living scores [17]. Al-Ani et al. have also described the benefits of early surgery. They found that patients had a greater chance of returning to independent living with a shorter length of hospital stay if they were operated on within 48 hours [9]. These findings are supported by the findings of a large prospective study by Siegmeth et al. [18].

The UK target proposed by NICE is to operate on the day of or the day after the presentation to the hospital. These guidelines serve to pragmatically mitigate morbidity and reduce mortality among these patients. Other bodies in the UK, such as BOA, also suggest that surgery should be performed within a 36-hour period and this is reflected in the Best Practice Tariff (BPT) [18]. As complication rates and length of admission increase, a greater economic burden is placed on the healthcare system in terms of managing these complications, placing further strain on already stretched services [19]. A Spanish study in 2013 by Etxebarria-Foronda et al. found that although a longer preoperative delay did not increase mortality, it was associated with higher overall costs [20]. A recent study by Chatziravdeli et al. reported that surgical delay of greater than 48 hours significantly prolonged admissions by up to five days in comparison to patients receiving surgery within 48 hours [21]. This translated to a greater overall cost per patient.

Our study findings focus on the time taken for patients to present to the hospital, which ultimately determines their waiting time for surgery. Previous studies have investigated pre-hospital management of lower limb fractures, albeit with a focus on analgesia and resuscitation [21]. As previously discussed, prolonged periods without adequate analgesia increase the risk of delirium and negatively impact the overall experience for patients. Therefore, focusing on analgesia and resuscitation is an appropriate strategy when treating NOFF patients in the pre-hospital setting. Ensuring efficient transfer and admission to the hospital should also be a priority [22]. Multiple variables can influence the timely transfer of NOFF patients to ED. These include ambulance availability, journey time, and environmental factors such as geographical location. Based on our literature review, there is minimal evidence of pre-hospital delays and strategies to improve this in the UK. Acknowledging this delay in routine assessment may reduce overall preoperative waiting time, incur a lower complication risk, and promote better outcomes.

There is also potential for pre-hospital delay for patients who live alone and are unable to call for help immediately after suffering an injury. Many studies describe difficulties faced by elderly patients to stand up following a fall, which is significantly worse in patients sustaining NOFF. Our study investigated this with respect to raw time data, but specific patient living circumstances were not accounted for. Hence, future studies can focus on factors that contribute to this delay and how it may be minimised. Fleming et al. have highlighted multiple community and social interventions such as alarms and support networks, which may be beneficial to this patient population [23].

It is widely accepted that performing timely surgery in NOFF patients promotes better outcomes. Our findings suggest that pre-hospital delay adds an extra 10% to the time a patient may wait for their surgery. In some cases, this delay can even account for a significantly larger proportion of time, with one patient experiencing a further 103% delay prior to hospital arrival. The time patients spend waiting to arrive at the hospital is often overlooked when considering surgical list planning. Our results suggest that this can vary from 59 minutes to 14 hours, and hence there is potentially a greater risk to some patients than what is initially apparent. This study is the first of its kind to be conducted in the UK, presenting unique data and highlighting a critical variable that must be carefully considered in the management of NOFF patients. We believe this to be an important factor that should be considered with respect to the surgical prioritisation of NOFF patients.

This study was observational in nature and has certain limitations. Firstly, the data only show timings and have not yet been correlated to outcomes. Secondly, the data were collected during the start of the coronavirus disease 2019 (COVID-19) pandemic. This could have potentially introduced confounding factors with regard to ambulance response times and ED limitations at the time, specifically contributing to reduced departmental capacity, social distancing, and increased ambulance wait time outside of the ED. However, it is likely that the NHS will continue to face these challenges for some time. Thirdly, our sample size was relatively small, leading to a higher chance of anomalous results that may skew our findings. However, despite the aforementioned limitations, the study provides much-needed insights into a topic that has been largely overlooked.

## Conclusions

Delays in surgery for NOFF patients lead to increased risk of mortality and morbidity. Pre-hospital time is often overlooked when analysing surgical delays, and this factor is likely to continue to have a significant impact given the increasing pressures on ambulance services. In other words, patients with a longer pre-hospital time are likely to be at greater risk of complications. Hence, we recommend that the pre-hospital delay be considered for all NOFF patients when prioritising assessment and the ordering of the surgical operating list. Further studies are needed to more comprehensively assess and quantify the effect of pre-hospital delays on morbidity and mortality.
